# Glomerular input patterns in the mouse olfactory bulb evoked by retronasal odor stimuli

**DOI:** 10.1186/1471-2202-14-45

**Published:** 2013-04-08

**Authors:** Yuichi Furudono, Ginny Cruz, Graeme Lowe

**Affiliations:** 1 Monell Chemical Senses Center, Philadelphia, PA, USA

**Keywords:** Retronasal olfaction, Olfactory bulb, Glomerulus, Optical imaging, SynaptopHluorin

## Abstract

**Background:**

Odorant stimuli can access the olfactory epithelium either orthonasally, by inhalation through the external nares, or retronasally by reverse airflow from the oral cavity. There is evidence that odors perceived through these two routes can differ in quality and intensity. We were curious whether such differences might potentially have a neural basis in the peripheral mechanisms of odor coding. To explore this possibility, we compared olfactory receptor input to glomeruli in the dorsal olfactory bulb evoked by orthonasal and retronasal stimulation. Maps of glomerular response were acquired by optical imaging of transgenic mice expressing synaptopHluorin (spH), a fluorescent reporter of presynaptic activity, in olfactory nerve terminals.

**Results:**

We found that retronasally delivered odorants were able to activate inputs to multiple glomeruli in the dorsal olfactory bulb. The retronasal responses were smaller than orthonasal responses to odorants delivered at comparable concentrations and flow rates, and they displayed higher thresholds and right-shifted dose–response curves. Glomerular maps of orthonasal and retronasal responses were usually well overlapped, with fewer total numbers of glomeruli in retronasal maps. However, maps at threshold could be quite distinct with little overlap. Retronasal responses were also more narrowly tuned to homologous series of aliphatic odorants of varying carbon chain length, with longer chain, more hydrophobic compounds evoking little or no response at comparable vapor levels.

**Conclusions:**

Several features of retronasal olfaction are possibly referable to the observed properties of glomerular odorant responses. The finding that retronasal responses are weaker and sparser than orthonasal responses is consistent with psychophysical studies showing lower sensitivity for retronasal olfaction in threshold and suprathreshold tests. The similarity and overlap of orthonasal and retronasal odor maps at suprathreshold concentrations agrees with generally similar perceived qualities for the same odorant stimuli administered by the two routes. However, divergence of maps near threshold is a potential factor in perceptual differences between orthonasal and retronasal olfaction. Narrower tuning of retronasal responses suggests that they may be less influenced by chromatographic adsorption effects.

## Background

Olfaction begins with the delivery of volatile organic compounds (odorants) to the olfactory epithelium where airborne molecules partition into the olfactory mucosa and are detected by olfactory receptors (ORs) expressed in olfactory sensory neurons (OSNs). Odorants can reach the epithelium by two distinct routes: an *orthonasal* route through the anterior nares, as occurs during inhalation and sniffing, and a *retronasal* route through the mouth, nasopharynx and posterior nares, as occurs during eating and drinking. Orthonasal stimuli convey critical information about the external world, such as the presence of potential dangers, mates or food sources, whereas retronasal stimuli represent sensory qualities of food and drink already ingested into the oral cavity.

In 1982, Rozin [1] first argued that olfaction is a dual sense, in which odor stimuli delivered via orthonasal and retronasal routes are processed and perceived differently. This idea has been investigated by several studies comparing orthonasal and retronasal olfaction in humans. Retronasal olfaction appears to be less sensitive, as indicated by psychophysical studies reporting lower performance in terms of thresholds [2-4], odor identification [5] or rated odor intensities in suprathreshold testing [6]. There is also evidence for qualitative differences in perceived odors. Subjects whose salivation reflex was habituated to orthonasally presented food odors still responded to the same odorants administered retronasally, and vice versa [7]. Speed and frequency of the swallowing reflex was facilitated by retronasal, but not orthonasal odors [8]. Direct measurements of brain activity have demonstrated that the two stimulation routes recruit different central pathways. Orthonasal and retronasal presentations of the same odorants evoked cortical event-related potentials with different amplitudes and latencies [2,9,10], and activated different brain areas visualized by functional magnetic resonance imaging [2,11,12].

Both central and peripheral mechanisms are thought to be involved in perceptual differences between orthonasal and retronasal odors. Central olfactory pathways responding to retronasal stimuli may be differently modulated by specific contexts of food odors, somatosensory localization of stimuli in the mouth, and integration of convergent gustatory signals [11-14]. In the periphery, weaker retronasal sensitivity may be a consequence of less efficient transport of odorants to receptors in the olfactory mucosa by reverse airflow. Computational modeling has predicted that expiratory airstreams do not penetrate the olfactory sensory region of the nasal cavity as effectively as inspiratory airstreams [15-17]. The electroolfactogram (EOG), a measure of the responses of OSNs in the nasal mucosa, was found to be smaller for retronasal than orthonasal stimuli in both humans [2,4] and rats [18]. Peripheral factors might also account for odor-specific differences between orthonasal and retronasal perception. Physical access and adsorption of odorants to olfactory vs. non-olfactory mucosa depends on the direction of airflow [19,20] and is influenced by physicochemical properties of odorants [21,22]. Mass spectrometry measurements showed that for retronasal delivery, hydrophilic odorants attained lower concentrations in the olfactory cleft than hydrophobic odorants [23]. These findings suggest a significant role for the physics of stimulus delivery in the periphery in establishing functional differences between orthonasal and retronasal olfaction.

To what extent are differences between orthonasal and retronasal olfaction determined by peripheral rather than central mechanisms? In order to isolate peripheral factors, it is critical to determine how the initial encoding of odors by ORs in the nose differs between the two routes of stimulus delivery. Individual OSNs express a single OR chosen from a repertoire of ~10^2^ – 10^3^ ORs [24-26]. Each OR is tuned to recognize multiple odorants bearing a specific range of molecular structural features [27,28], and each odorant is encoded by a unique combination of ORs [29,30]. However, various ORs are not uniformly expressed across the nasal mucosa, but occur in restricted horizontal zones [31-33]. This means that odorants with different adsorption patterns for orthonasal vs. retronasal delivery might activate different populations of ORs which could transmit different odor representations to the brain. We investigated this possibility by mapping and comparing OR codes for odorant stimuli delivered via orthonasal and retronasal routes.

Mapping of OR codes is facilitated by the precise topographic organization of olfactory sensory afferents, in which OSNs expressing the same OR send convergent projections to a few glomeruli at stereotypic locations on the surface of the main olfactory bulb [34-36]. Combinatoric activation of ORs generates odorant-specific spatial patterns of glomerular input (‘odor maps’) [37] that can be partially visualized in animal preparations by optical imaging of the dorsal olfactory bulb [38-42]. Imaging is facilitated by transgenic mice engineered to express synaptopHluorin (spH), a fluorescent indicator of presynaptic activity [43], in OSN terminals innervating olfactory glomeruli [44]. Using these mice, we compared OR encoding of orthonasal and retronasal stimuli delivered under controlled flow conditions. We found that retronasal stimuli evoked weaker glomerular activation with higher thresholds. Maps of glomerular input were sparser, and individual glomerular responses more narrowly tuned when screened with homologous series of odorants. These observations suggest that peripheral factors play an important role in determining the lower sensitivity of retronasal olfaction seen in psychophysical tests. We also detected glomeruli with retronasal, but not orthonasal responses at threshold, which could potentially encode distinct retronasal odor qualities. While this manuscript was in preparation, another study mapping retronasal glomerular responses in the rat appeared [45]. Similar findings were reported, suggesting that our results can be extrapolated across different mammalian species.

## Results

Analysis and comparison of the peripheral determinants of odor coding in orthonasal and retronasal olfaction is challenging because of their complex and different stimulus dynamics. Orthonasal odorants are conveyed to the olfactory epithelium by varied sampling behaviors that can range from passive, low frequency (~ 1 – 3 Hz) inhalation locked to the respiratory cycle, to active high frequency (~ 3 – 12 Hz) bouts of investigative sniffing [46,47]. In contrast, retronasal odorants released in the mouth are transported to the nasal cavity over longer time scales of many seconds by coordinated movements of the soft palate and pharynx during food chewing and swallowing [48,49]. In both cases OR responses could conceivably be influenced by complicated airflow patterns and dynamics that have not been fully characterized [15]. In particular, it is unclear how to introduce odorants retronasally to precisely mimic natural olfactory stimulation during feeding. Considering these limitations, we have addressed a simpler question – are there differences in OR activation and odor encoding for orthonasal and retronasal routes when odorants are delivered by steady airflow in forward and reverse directions through the nasal cavity? This approach has the technical advantage of easily reproducible, well controlled flow conditions with readily quantifiable velocity and stimulus parameters. For our study, steady airflow stimulation was a good first order approximation to more complex pulsatile stimulation because spatial patterns and dose–response relations of OR glomerular input, as measured by presynaptic imaging, depend mainly on maximal flow rate, and not on phasic sniff frequency [50]. Moreover, computational modeling analysis of airflow in the rodent nasal cavity has shown that over a physiological range of sniff frequencies (2 Hz – 12 Hz), steady flow is a valid approximation over 84% – 72% of the sniff cycle in both orthonasal and retronasal directions [17]. We therefore employed steady flow as a first step towards comparing and contrasting the abilities of odorants to penetrate the olfactory mucosa via orthonasal or retronasal routes of entry to elicit distinctive odor encoding patterns of glomerular input.

### Retronasal responses are smaller and slower than orthonasal responses

**Figure 1:**
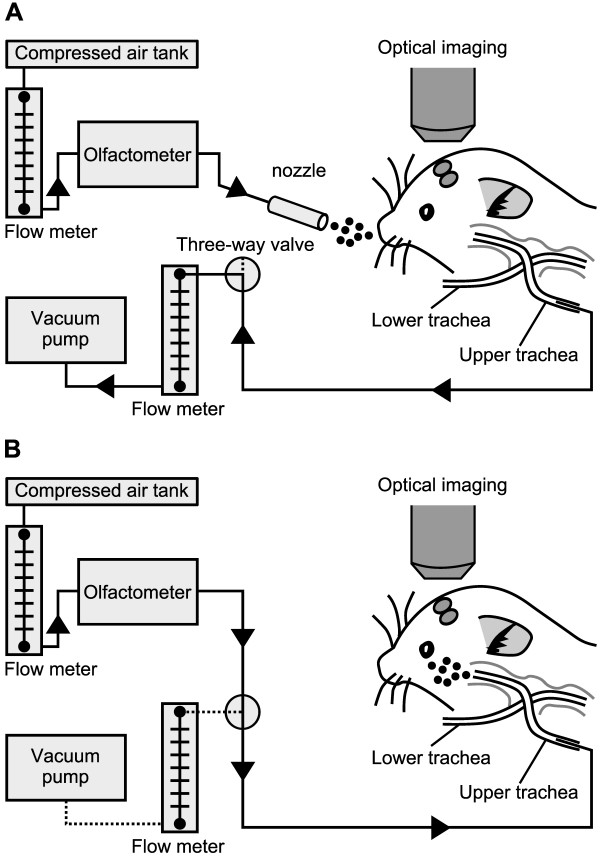
**Schematic diagrams of the odor presentation system. A:** Experimental set-up for orthonasal odor presentation. The olfactometer was connected to an odor nozzle and presented odor stimuli to the mouse’s nose. The upper cannula was connected to a flow meter and a vacuum pump, which controlled nasal airflow rates. The lower cannula accessed the lower trachea and remained open for normal breathing. **B:** Experimenatal set-up for retronasal odor presentation. The olfactometer was connected to a three-way valve and presented odor stimuli through the upper cannula. Our set-up permitted reciprocal switching of orthonasal and retronasal delivery modes in the same animal.

**Figure 2:**
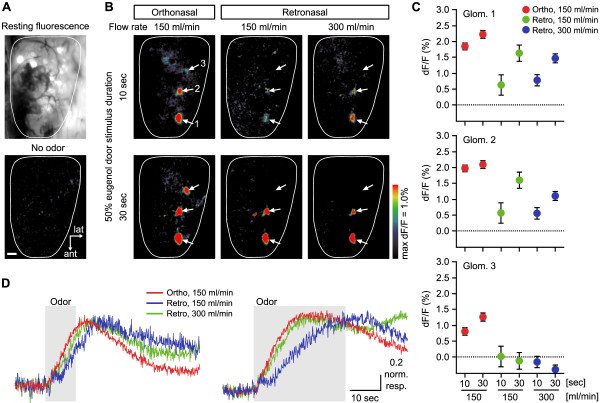
**Odor-evoked spH responses to retronasal airflow in the dorsal olfactory bulb. A:** Resting fluorescence image of the dorsal olfactory bulb showing spH-labeled glomeruli visible through thinned bone. No glomerular responses were observed in the absence of odor stimuli. Scale bar = 200 μm. **B:** Glomerular response maps to 50% v/v eugenol presented orthonasally or retronasally under different flow rates and stimulus durations. The pseudocolored images show % change in spH fluorescence from resting fluorescence intensity recorded before stimulus onset. Images were obtained from the same animal preparation. The three numbered white arrows indicate the glomeruli for which odor-evoked responses were analyzed in panels C and D. **C:** The amplitudes of orthonasal and retronasal responses at different flow rates and stimulus durations. Response amplitudes were quantified for glomeruli #1 – #3 corresponding to numbered arrows in panel B. Each value is the mean ± SE of 3 trials. **D:** Normalized response traces evoked by 10 s (left) and 30 s (right) stimulus pulses in glomerulus #1. The shaded regions indicate the duration of odor pulses. Red lines indicate orthonasal response traces at a flow rate of 150 ml/min. Blue and green lines indicate retronasal response traces at 150 and 300 ml/min, respectively.

First, we measured orthonasal and retronasal odorant responses of individual glomeruli to 50% v/v eugenol, delivered by double tracheotomy (Figure 1) at 150 and 300 ml/min flow rates, with 10 or 30 s stimulus durations. Our tested flow rates equal or exceed estimated peak flow rates (~120 ml/min) for strong inhalations or exhalations by sniffing mice [50,51]. This allowed us to acquire maximal patterns of OR activation that should include all physiologically responsive glomeruli. Our stimulus durations of > 10 s roughly match the slow time scales of spH responses to odorants, reflecting the sustained exocytosis and recycling of presynaptic vesicles in OSN terminals. The extended durations also enabled temporal integration of spH responses, which greatly improves the signal-to-noise ratio for detection of weakly activated glomeruli [44], allowing us to acquire more complete odor maps for making comparisons. Figure 2 shows that increments in spH fluorescence were detected in overlapping sets of glomeruli during both orthonasal and retronasal stimulation. The overlap was incomplete: glomeruli #1 and #2 (Figure 2B, lower arrows) were activated in both stimulation modes, whereas glomerulus #3 (Figure 2B, upper arrow) showed no retronasal response under any stimulus condition. We quantified response amplitudes (ΔF/F) of glomeruli #1 – #3 to compare their responsiveness to orthonasal and retronasal stimulation (Figure 2C). As indicated in the odor maps, glomerulus #3 did not respond to any retronasal stimuli, but did exhibit orthonasal responses. Glomeruli #1 and #2 responded to both orthonasal and retronasal stimulation in a similar manner, with similar potency. We conducted statistical analyses of the combined response amplitude data. The orthonasal response was approximately 3-fold greater than the retronasal response under the same stimulus condition of 150 ml/min and 10 s (1.92 ± 0.09 vs. 0.60 ± 0.21, *t* = 9.56, *p* < 0.05). Prolonging stimulus duration to 30 s had no significant effect on orthonasal response amplitudes (10 s vs. 30 sec: 1.92 ± 0.09 vs. 2.16 ± 0.09, *t* = 1.35, *p* = 0.23, NS). To assess the effects of flow rate and stimulus duration on retronasal responses, we performed two-way analysis of variance. A significant effect of stimulus duration was detected (*F*_1, 20_ = 24.58. *p* < 0.05), and no flow rate and stimulus duration × flow rate interaction effects were detected. The statistical results indicate that stimulus duration, rather than airflow rate, contributed to increased retronasal response amplitudes. This is consistent with the boosting of weak spH signals by a longer period of temporal integration [44]. Figure 2D shows time courses of normalized spH signals from glomerulus #1. At both stimulus durations, retronasal responses at 150 ml/min exhibited longer rise times than orthonasal responses, and their rise times were shortened when flow rate was stepped up to 300 ml/min. The retronasal responses also exhibited recovery time courses that were slower than those of orthonasal responses. Thus, retronasal stimulation evoked smaller responses with slower kinetics for odor stimuli of the same duration presented at the same concentration.

### Retronasal stimuli are less effective than orthonasal stimuli in evoking glomerular responses

**Figure 3:**
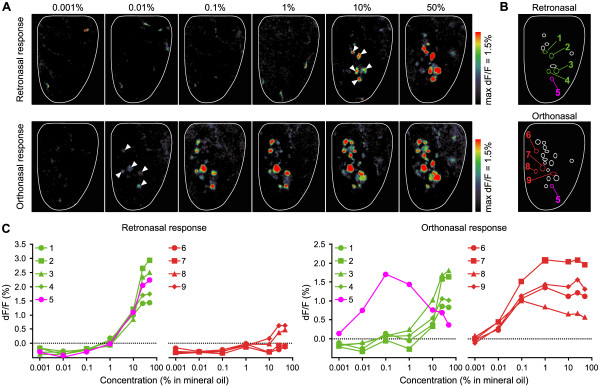
**Comparison of dose–response relationships for retronasal and orthonasal airflow. A:** Odor maps from a single mouse, elicited with increasing concentrations of valeric acid (0.001% – 50% v/v). Retronasal stimuli were presented for 30 s at 300 ml/min (upper panels). Orthonasal stimuli were delivered for 10 s at 150 ml/min (lower panels). White arrow heads indicate glomeruli that exhibited responses at near-threshold concentrations for each airflow pathway. Scale bar = 200 μm. **B:** Spatial arrangement of activated glomeruli. Open circles show the positions of glomeruli whose responses were increased by 0.5% from resting fluorescence. Among open circles, numbered and colored circles indicate the numbered glomeruli that exhibited either or both retronasal and orthonasal responses at near-threshold concentrations in panel A (retronasal: green, #1 – #4; both: magenta, #5; orthonasal: red, #6 – #9). **C:** The dose–response relationship to retronasal (left) and orthonasal (right) odor. The response amplitudes were analyzed for glomeruli #1 – #9 that correspond to the number in panel B. Each value is the mean of 3 trials. Note: the apparent hotspot in upper right quadrant of 0.001% v/v retronasal map was a spurious signal from a blood vessel.

We next compared response thresholds for orthonasal and retronasal stimulation routes using another test odorant, valeric acid, which evoked retronasal responses in a greater number of glomeruli. Orthonasal stimuli were delivered for 10 s at 150 ml/min, and retronasal stimuli for 30 s at 300 ml/min. The longer duration and higher flow rates were necessary to boost the weaker retronasal responses so that we could chart their dose–response relations. Over ascending concentration series with one log unit dilution step, glomerular responses to retronasal stimulation were first detected at 10% v/v concentration, and orthonasal responses at 0.01% v/v (Figure 3A). Increasing the odorant concentration recruited signals from additional glomeruli in both stimulation modes. Orthonasal stimulation activated a total of 19 glomeruli over the tested series of concentrations, with 10 overlapping glomeruli also activated by retronasal stimulation. Glomeruli responding at threshold for each airflow route are indicated by white arrow heads, and positions of activated glomeruli are outlined on schematic maps as open circles (Figure 3B). Numbered colored circles show glomeruli responding to one or both routes at their response thresholds. Only one glomerulus was responsive to both routes (glomerulus #5, magenta circle) at threshold. The remaining glomeruli responded to either orthonasal or retronasal stimulation at each threshold (retronasal: green, #1 – #4; orthonasal: red, #6 – #9). Figure 3C shows the dose–response relations of identified glomeruli. Glomeruli #1 – #4 exhibited retronasal responses at 10% v/v and their response amplitudes increased at higher concentrations. These glomeruli responded to both orthonasal and retronasal stimulation with similar sensitivity. Glomeruli #6 – #9 exhibited orthonasal responses at 0.01% v/v and their responses were saturated at 0.1 or 1% v/v, indicating that they have higher sensitivities to valeric acid than glomeruli #1 – #4. Nevertheless, glomeruli #6 – #9 showed poor responsiveness to retronasal stimulation. Two of these (#6, #7) were completely insensitive to retronasal stimulation over the entire concentration range. The remaining 2 glomeruli (#8, #9) exhibited weak retronasal responses at high concentrations. Glomerulus #5 was unique in that it exhibited retronasal responsiveness similar to #1 – #4, and was also sensitive to orthonasal stimulation, with a large response at 0.01% v/v that fell off as concentration exceeded saturation. Thus, different airflow routes activated different subsets of glomeruli at their respective response thresholds. Moreover, some glomeruli showed no detectable retronasal responses even though they had high orthonasal sensitivity to the test odorant. These results indicate that the retronasal route is generally less effective in evoking glomerular responses than the orthonasal route, even though retronasal stimuli were presented for longer durations at higher flow rates. This difference would be more pronounced at equalized stimulus durations and flow rates, because shortening the duration decreases the response (Figure 2C).

### Retronasal odor maps are subsets of orthonasal odor maps

**Figure 4:**
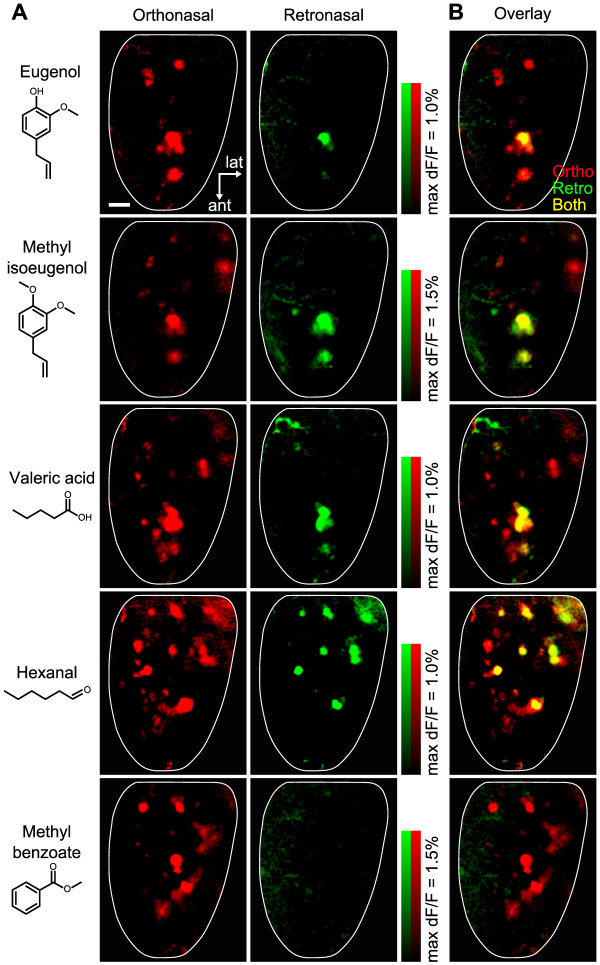
**Overlap between orthonasal and retronasal response patterns. A:** Glomerular activity patterns of 5 different odorants presented orthonasally or retronasally in the same animal. Eugenol (50% v/v for both stimulation modes), methyl isoeugenol (50% v/v for both), valeric acid (50% v/v for both), hexanal (0.1% v/v for orthonasal, 10% v/v for retronasal), methyl benzoate (50% v/v for both) were used for spH imaging. Orthonasal stimuli were delivered for 10 s at 150 ml/min. Retronasal stimuli were delivered for 30 s at 300 ml/min. Response maps for each stimulus mode are rendered in different colors (orthonasal: red, retronasal: green). Scale bar = 200 μm. **B:** Overlay of orthonasal and retronasal representations expressed in different color channels to indicate the glomeruli responding to one or both airflow pathways (orthonasal only: red; retronasal only: green; both: yellow).

Some glomeruli failed to respond to odorants delivered retronasally as shown in Figures 2 and 3. To confirm this for a wider range of odorants, we compared in the same animal the glomerular patterns of orthonasal and retronasal response to 5 different odorants. Stimuli were applied at high suprathreshold concentrations to activate many glomeruli. Figure 4A shows that all tested odorants evoked orthonasal responses in distinct but partly overlapping sets of glomeruli. In contrast, no retronasal response was detected for methyl benzoate, and other test odorants activated a smaller number of glomeruli when presented retronasally than orthonasally. In Figure 4B, spatial patterns of orthonasal and retronasal responses are overlaid to indicate glomeruli responding to one or both airflow routes (orthonasal only: red; retronasal only: green; both: yellow). Red and yellow hotspots in merged images indicated that some glomeruli were selective for orthonasal airflow (red) and other glomeruli had no selectivity for airflow pathways (yellow). There were no retronasal-specific glomeruli, as indicated by the absence of green hotspots for all test odorants. These data show that, although orthonasal and retronasal odor maps could be quite different at their respective thresholds (Figure 3), when fully activated by strong suprathreshold stimuli the retronasal maps were generally sparser than orthonasal maps, including only subsets of the glomeruli responding to the same orthonasal odorant.

### Retronasal responses are more narrowly tuned to homologous series of odorants

**Figure 5:**
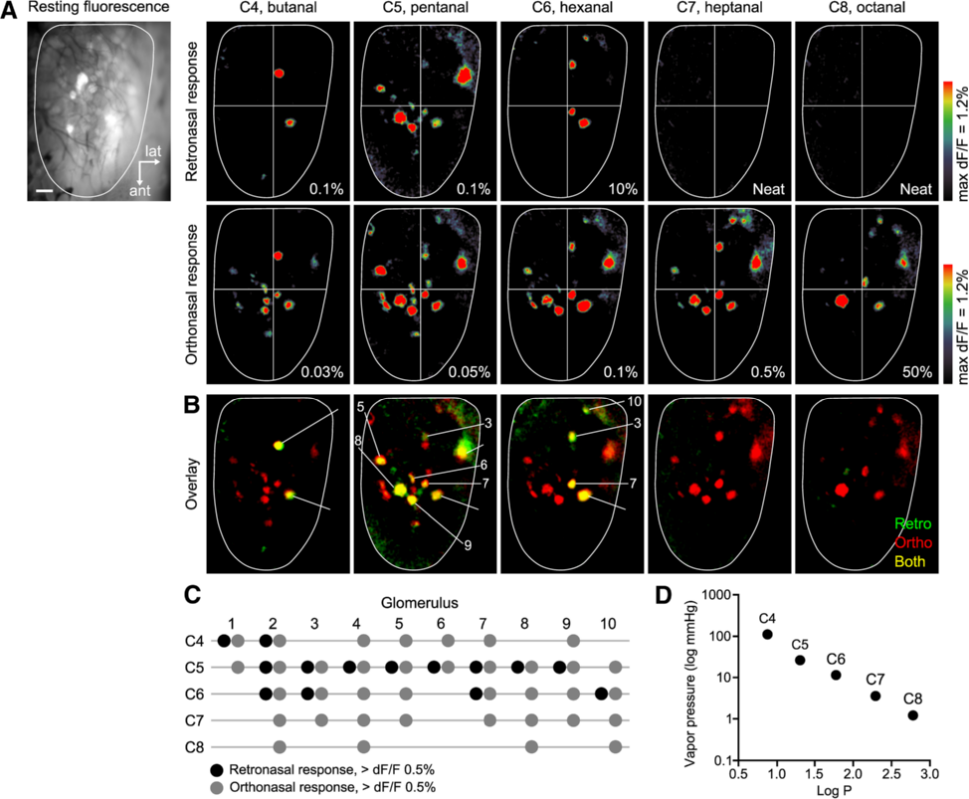
**Glomerular activity patterns of aliphatic aldehydes presented by the retronasal or orthonasal pathway. A:** Response maps evoked by a homologous series of aliphatic aldehydes (C4 – C8) from a single mouse. The left-most panel shows the resting fluorescence image. Scale bar = 200 μm. Retronasal stimuli were delivered for 30 s at 300 ml /min (upper panels). Orthonasal stimuli were delivered for 10 s at 150 ml /min (lower panels). Percent dilutions in mineral oil are given in each panel. **B:** Overlay of retronasal and orthonasal response maps expressed in different color channels to indicate glomeruli responding to one or both airflow pathways (retronasal only: green, orthonasal only: red, both: yellow). **C:** Dot-plot representation of responses of the glomeruli numbered in panel B. Dots correspond to > 0.5% increase in response amplitudes. Black and gray filled circles show retronasal and orthonasal responses, respectively. **D:** Vapor pressure and log P of test aldehydes (log P = index of hydrophobicity).

It has long been thought that odor perception is affected by differential access and adsorption of odorants in the olfactory epithelium that depend on their physicochemical properties [52]. For example, polar compounds may be more readily adsorbed by the aqueous mucosa and removed earlier in the airflow stream, whereas hydrophobic compounds may be transported further downstream and dispersed more uniformly [53,54]. These chromatographically imposed deposition patterns could interact with inherent OR expression patterns, resulting in differences between OR encoding of odorants delivered by orthonasal vs. retronasal routes [19,20,22,55]. Electroolfactogram recordings appeared to confirm that some non-polar (hydrophobic) odorants have greater retronasal access to local sites of olfactory mucosa than polar odorants [18]. To examine the impact of physicochemical properties on OR encoding of orthonasal vs. retronasal stimuli, we mapped glomeruli activated by a homologous series of aliphatic aldehydes with variable chain lengths ranging from 4 to 8 carbon atoms. As these compounds are structurally related, their glomerular responses in the dorsal olfactory bulb are strongly overlapped [38,41]. This allowed us to compare molecular tuning of many individual glomeruli over a range of odorant hydrophobicity, as the longer chain aldehydes have higher values of log P (log octanol/ water partition coefficient, an index of hydrophobicity) (Figure 5D).

Our initial attempts to map and compare responses to the different aldehydes were hindered by difficulties in detecting much weaker retronasal responses at similar applied concentrations (Figure 3), and by large differences in volatility of compounds with different carbon chain lengths (Figure 5D). To overcome these difficulties, we delivered retronasal stimuli with a longer duration and higher flow rate (30 s, 300 ml/min) to boost weak spH responses into the range of detectability. We then tested liquid dilutions at higher concentrations for aldehydes with longer carbon chains, to offset their lower volatilities. Dilutions were empirically adjusted to yield robust and consistent responses from multiple glomeruli through 3 consecutive trials. These steps enabled us to detect and identify a number of responsive glomeruli for comparison across carbon chain lengths under both orthonasal and retronasal stimulation.

Figure 5A shows a series of orthonasal and retronasal odor maps for C4 – C8 aldehydes with ascending concentrations in mineral oil. For orthonasal stimulation, all tested aldehydes were able to elicit strong responses from many glomeruli in highly overlapping patterns. On the other hand, retronasal responses could only be seen over carbon chain lengths C4 – C6, and were undetectable for longer chain aldehydes (C7 – C8) even when maximal concentration (neat) stimuli were presented. In Figure 5B, an overlay of retronasal (green) and orthonasal (red) maps shows that retronasal responses were universally more sparse, i.e., they were subsets of orthonasal-responsive glomeruli. There were no green glomeruli responding only to retronasal input, at any carbon chain length. We identified 10 glomeruli responding to retronasal stimuli and analyzed their response profiles for both stimulus routes (Figure 5C). Individual glomeruli displayed orthonasal responses to 2 – 5 aldehydes with consecutive carbon chain lengths. In contrast, their retronasal responses were restricted to a narrower range of 1 – 3 aldehydes.

**Figure 6:**
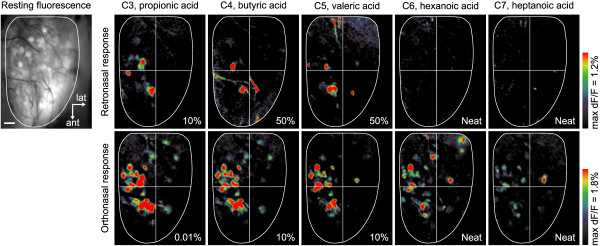
**Mapping of glomerular responses to aliphatic acids (C3 – C7).** Odor maps were obtained from a single mouse. Retronasal stimulus was delivered for 30 s at 300 ml/min (upper panels), orthonasal stimulus for 10 s at 150 ml/min (lower panels). Percent dilutions in mineral oil are given in each panel. The resting fluorescence of olfactory bulb is shown (leftmost panel). Scale bar = 200 μm. No retronasal responses were found for hexanoic acid and heptanoic acid.

In the maps shown in Figure 5A, it appeared that the effective molecular tuning of ORs for retronasal stimulation is narrower than for orthonasal stimulation. To confirm this, it was necessary to account for odorant concentration differences among the maps. We noted that C5 (pentanal) yielded the strongest responses to both orthonasal and retronasal inputs, with greatest overlap between them (8/10 glomeruli). To compare molecular tuning characteristics, we estimated orthonasal and retronasal vapor levels at the tested liquid dilutions by interpolating published measurements [56], and normalized them relative to the respective C5 values (i.e. at 0.05% v/v or 0.1% v/v). Estimated ratios of vapor levels relative to C5 were: retronasal, C4 2.31: C5 1.00 : C6 1.49: C7 0.89: C8 0.67; orthonasal, C4 2.00: C5 1.00: C6 0.055: C7 0.068: C8 1.15. This showed that C4, C6 and C7 vapor levels relative to C5 were all higher in the retronasal case, and if reduced to match orthonasal levels the corresponding retronasal maps would be sparser than those in Figure 5A. The C8 vapor level relative to C5 was somewhat larger in the orthonasal case (1.15 vs. 0.67), and would be equalized by reducing C8 from 50% v/v to 25% v/v [56]. This may make the orthonasal C8 map somewhat sparser, but not less so than the retronasal map which lacks any responsive glomeruli. Finally, we note that if relative vapor levels were equalized across carbon chain lengths to match C5 = 1.00, orthonasal responses would be either little changed, or boosted (C6 and C7 elevated), and retronasal responses either little changed or slightly reduced. Thus, our data support the conclusion that effective molecular tuning of glomeruli across homologous series of aldehydes is narrower for retronasal than orthonasal stimulation. In particular, the ability of retronasal stimuli to activate glomeruli was more sensitive to increasing carbon chain length, as shown by the failure to evoke any C7 or C8 responses, even with neat odorants. Similar results were obtained with a homologous series of aliphatic acids (C3 – C7, Figure 6). Relative tuning was similar over C3 – C5, but acids with longer carbon chains (C6 – C7) evoked no retronasal responses even at neat concentrations, in contrast to the clear responses evoked by orthonasal delivery at the same concentration.

## Discussion

In this study we applied optical imaging to spH mice to compare the patterns of presynaptic OR inputs received by glomeruli in the dorsal olfactory bulb evoked by orthonasally and retronasally applied odorants. We found that retronasal stimuli were less effective in eliciting glomerular responses than orthonasal stimulation, as measured by response amplitudes and thresholds. Retronasal stimuli were also more sparsely coded since they activated subsets of orthonasal responsive glomeruli. Mapping responses to homologous series of aliphatic aldehydes and acids revealed a narrower effective molecular tuning for retronasally delivered odorants.

These properties of the retronasal responses must be determined by peripheral factors, because presynaptic spH signals represent the summated responses of OSNs residing in the olfactory epithelium [44]. Glomeruli receive inputs from OSN populations expressing unique ORs [36] dispersed across overlapping zones of sensory epithelium lining the turbinates and septum of the nasal cavity [31,32]. The response of each glomerulus will depend on the ligand specificity of the associated OR, and the concentrations of odorants reaching sites of OR expression. Odorant ligand tuning is an intrinsic property of the receptor, independent of airflow direction, so differences between orthonasal and retronasal responses must be due to differential access of odorants to receptors. We found significantly higher thresholds, smaller amplitudes and longer rise times for retronasal glomerular responses to a variety of odorants delivered at the same concentrations, flow rates and durations. This is consistent with local electroolfactogram recordings in the rat olfactory epithelium that revealed smaller retronasal responses with longer latencies [18]. It implies that fewer odor molecules reached OR binding sites by the retronasal route, compared to the orthonasal route. One possible explanation for reduced access could be a difference between the forward and reverse airflow patterns. Computational fluid dynamics simulations of the rat nasal cavity have revealed more restricted flow to parts of the olfactory epithelium during expiration, and the calculated distributions of odorant adsorption are different for inspiration vs. expiration. Computed orthonasal airflow is characterized by S-shaped streamlines transporting odorants to dorsomedial or dorsolateral zones of the nasal cavity [15,17] where there is selective expression of ORs projecting to glomeruli located on the dorsal part of the olfactory bulb [32,57,58]. During retronasal airflow, S-shaped streamlines are predicted to be much weaker, so that odorized air will be diluted more, and will deposit fewer odorant molecules in the dorsal epithelial zone [54]. Because our spH imaging method can only inspect the dorsal surface of the bulb, the glomeruli that we studied would mostly receive inputs from ORs located in dorsal zones of epithelium that are exposed to higher odorant concentrations under orthonasal stimulation, and to lower concentrations under retronasal stimulation.

Stimulus concentration gradients that result from the dilution of odorized air entering the nasal cavity in either orthonasal or retronasal directions may interact with spatial patterns of OR zonation to shape glomerular input maps. This may be a factor in the different orthonasal and retronasal patterns of glomerular activation evoked by valeric acid at threshold (Figure 3). If different ORs occupy different spatial zones in forward and reverse concentration gradients, then apparent glomerular thresholds could change when airflow direction is switched. In Figure 3, it appears that the red glomeruli (#6 – #9) may receive input from OR zones that are well stimulated by orthonasal flow, and the green glomeruli (#1 – #4) input from OR zones that are better positioned for sampling retronasal flow; the magenta glomerulus (#5) seems to occupy an intermediate position.

Differential attenuation of orthonasal vs. retronasal stimuli might depend not only on flow dilution, but also on odorant adsorption. Concentration gradients will be accentuated if stimulus molecules are removed by adsorption to upstream mucosal surfaces while being transported to downstream receptor sites (chromatographic hypothesis) [19,22,55,59]. Previous studies suggested that initial adsorption of odor molecules to non-olfactory regions such as the nasopharynx diminishes the amount of odorant entering the posterior nares and reaching the olfactory epithelium via the retronasal route [6,18,23,60]. Adsorption gradients of odorants are predicted to depend on their physicochemical properties, with steeper gradients generated by stronger mucosal adsorption of more polar compounds [23,53,60]. For retronasal delivery, electroolfactogram recordings of OSN responses [18] and direct mass spectrometry measurements [23] indicated that more polar odorants attain lower concentrations in the olfactory cleft and olfactory epithelium than hydrophobic odorants. Conversely, more hydrophobic odorants are predicted to have weaker adsorption and better retronasal penetration of the olfactory epithelium. However, the glomerular responses evoked by structurally related aliphatic aldehydes or acids applied at similar vapor levels revealed an opposite trend, i.e. the more hydrophobic, longer chain molecules evoked much weaker or no retronasal responses, in contrast to strong responses of their shorter, less hydrophobic homologs. In the retronasal case, C7 responses were undetectable at nearly the same estimated vapor level (~90%) as C5, which strongly activated 8 glomeruli; in the orthonasal case C8 only activated 4 glomeruli at an estimated vapor level 15% higher than C5, which more strongly activated 10 glomeruli. This would indicate that the molecular tuning is largely determined by coarse chemotopic organization of intrinsic OR tuning in the periphery, which is mapped to glomerular domains of the bulb. Systematic studies have shown that glomeruli tuned to carbon chains of increasing length are located progressively further away from the dorsal region, towards the medial/ lateral and ventral domains [61]. Lower intrinsic sensitivities of dorsal glomeruli to longer aliphatic chains could explain the failure of C7 and C8 odorants to evoke detectable responses after their vapor levels are diluted by weak retronasal airflow to the dorsal epithelium. These same compounds could still evoke clear responses during orthonasal presentation because higher odorant concentrations were transported to the dorsal epithelium. Supposing that dorsal zone ORs and their glomeruli are intrinsically better tuned for hydrophilic over hydrophobic odorants, any chromatographic adsorption gradients would tend to counteract this by favoring transport of the more hydrophobic odorants and broadening effective molecular tuning. Thus, our finding of narrower tuning for retronasal stimulation suggests adsorption effects in retronasal airflow were less effective than for orthonasal airflow. This seems to differ from human studies indicating stronger retronasal adsorption of more polar odorants [23,60]. However, direct comparison of mouse and human data is complicated by differences between the response assays, anatomy of adsorption pathways, and tracheal vs. oral delivery of retronasal stimuli.

Our observations on encoding of retronasal odors by glomerular inputs in the mouse are similar to results recently reported by Gautam & Verhagen (2012) in the rat olfactory bulb [45]. They also found overlap of orthonasal and retronasal odor maps of dorsal glomeruli, and their retronasal responses were also smaller and slower than orthonasal responses. Odorants with higher vapor pressure were more effective in eliciting retronasal responses, consistent with our experiments in which less volatile odorants required higher concentrations in mineral oil to elicit robust retronasal responses. One difference was that they concluded that retronasal responses largely consist of the same set of activated glomeruli as orthonasal responses, while our data (Figures 3, 4 and 5) indicated that some glomeruli activated by orthonasal stimulation were non-responsive to retronasal stimulation by the same odorant, even though a higher concentration was applied with a longer duration. Their use of calcium imaging yielded recordings with sufficient temporal resolution to detect differences of ~100 ms in onset and times to peak, which could not be seen in our slower spH responses. They found longer delays in their retronasal responses to less hydrophobic compounds, suggesting that differential adsorption of polar odorants can fine tune the early dynamics of retronasal responses (< ~ 100 ms), which are relevant for fast temporal mechanisms of olfactory coding [62-64]. On the other hand, spatial maps of spH response integrated over longer periods (10 –30 s) may be relevant for modeling odor coding during the slow, sustained process of retronasal smelling that occurs during ingestion, chewing and swallowing of food.

The smaller amplitudes and lower sensitivities that we see in retronasal responses are consistent with psychophysical reports of higher thresholds and lower rated odor intensities for retronasal vs. orthonasal olfaction [2-4,6]. They suggest that the factors affecting peripheral neural coding could partly account for these perceptual differences. The higher threshold of glomerular responses means that the same odorants need to be introduced at higher concentrations retronasally to be detected by ORs in the olfactory epithelium. Lower ratings of perceived intensity could be related either to reduced strength of OSN inputs, or to sparser input maps. Odorant concentration and odor intensity may be encoded at the cellular level by spike rate or spike latency patterns of olfactory bulb output neurons [65-68], which depend on strengths of OSN inputs, or at the systems level by concentration-dependent recruitment of multiple glomeruli in odor maps [69]. The sparser retronasal maps might be expected to reduce glomerular overlap for different odorants, and hence enhance retronasal odor discrimination. However, performance in identifying trained odors is actually lower for retronasal olfaction [5], so potential enhancements may be outweighed by lowered sensitivity.

Did we find differences between peripheral neural encodings of orthonasal and retronasal stimuli that could potentially contribute to different perceived odor qualities, as originally suggested by Rozin in his duality hypothesis [1]? Retronasal odor maps differed in that they typically only included subsets of glomeruli responding to orthonasal stimulation. Whether this sparsening of maps would lead to perceptual differences depends on how central circuits process the changing input patterns. Maps normally become sparser at lower odorant concentrations as less sensitive glomeruli drop out of representations [40,70-72]. Odor quality may be invariant under smaller shifts in concentration [73,74] for some odorants [75], but could conceivably change with larger shifts for others [76,77]. Different peripheral mechanics of odorant flow dilution and adsorption, combined with spatially restricted OR expression zones, may cause glomeruli to drop out of retronasal maps in a different order than orthonasal maps, so that retronasal maps may no longer be subsets of orthonasal maps at their respective thresholds. For example, the white arrowheads in Figure 3 highlight quite different threshold maps for valeric acid with only one overlapping glomerulus (#5), and perhaps these maps encode different odor qualities. We speculate that further differences are likely to emerge from mapping responses of more ventrally located glomeruli, as these are likely to receive inputs from ventral zone ORs situated closer to the retronasal airstream. It will be important as well to consider whether perceptual interpretations of different retronasal and orthonasal odor maps might be influenced by their temporal properties, such as timing of glomerular inputs relative to sniffing. Olfactory sensory discrimination can be sensitive to sniff phase [78], and odor signals of retronasal origin might be identifiable by synchronization with the exhalation phase of the sniff cycle.

Any functional interpretations of our data should be qualified by the fact that our view of the peripheral odor code was restricted to the dorsal olfactory bulb. It is possible that some odorants might be more effectively detected by glomeruli located in more lateral or ventral domains of the bulb. These domains would receive inputs from ORs expressed in lateral or ventral zones of epithelium closer to the reverse airstream entering the internal nares. In electrophysiological recordings, retronasal responses in both epithelium and bulb were greater on the lateral side than on the dorsal side [18]. Retronasal stimuli might also be more efficiently detected by the septal organ of Masera, a small patch of sensory epithelium on the nasal septum adjacent to the nasopalatine duct, separate from the main olfactory epithelium [79]. Computational modeling showed that both inspiratory and expiratory airstreams have good access to the septal organ [15-17]. However, glomeruli receiving septal organ input are clustered on the ventro-medial surface of the bulb [80] which was not sampled by our imaging technique.

Retronasal odor signals are integrated with other oral sensory inputs, including taste and chemesthesis, to create sensations of flavor. There is a growing interest in retronasal olfaction for understanding flavor perception. Although most studies on retronasal olfaction have been conducted in humans, behavioral studies have also indicated the importance of the retronasal pathway in rodents [81-84]. Animal models permit broader experimental approaches for further investigating retronasal olfaction, especially neural mechanisms underlying the encoding of complex odorant mixtures, such as those released in the oral cavity that contribute to flavor and enjoyment of food.

## Conclusions

In the dorsal domain of the mouse olfactory bulb, olfactory receptor inputs to glomeruli can be activated by retronasal odorant stimuli. However, retronasal input is weaker and slower with higher thresholds than orthonasal input at comparable vapor levels and flow rates. Retronasal odor maps are sparser and typically comprised of subsets of orthonasal maps. At threshold, the maps can be more distinct with little overlap, suggesting a possible means for encoding different perceptual qualities. Retronasal responses are more narrowly tuned to carbon chain lengths of odorants, and are attenuated for longer chain, more hydrophobic compounds. This suggests that retronasal maps are less influenced by differential adsorption effects. The peripheral olfactory system appears well adapted to serve dual functions: rapid, sensitive orthonasal responses enable quick sampling and detection of critical odor signals at low concentrations in the external environment; slower, higher threshold retronasal responses are more appropriate for sensing high concentration volatiles released from food in the mouth, for multisensory integration and flavor perception. A more complete picture of peripheral coding in retronasal olfaction awaits detailed mapping of glomerular responses in other domains of the bulb.

## Methods

### Animals and surgery

Experiments were performed on heterozygous spH mice (B6;129P2-*Omp*^*tm2(spH)Mom*^/J × B6129 wild type) of both sexes, ranging from 9 to 15 weeks old. Wild type and mutant breeder mice were obtained from The Jackson Laboratory (Bar Harbor, Maine) and colonies were established and maintained in-house. All animal procedures were approved by the Institutional Animal Care and Use Committee at the Monell Chemical Senses Center. Mice were anesthetized by intraperitoneal injection of ketamine (100 mg/kg) and xylazine (10 mg/kg), and atropine (5 mg/kg) was injected subcutaneously. A double tracheotomy was performed to allow control of odorant access to the nasal cavity. A cannula was inserted through the upper trachea to rest over the soft palate. The mice breathed freely through the lower tracheotomy tube. Mice were secured on a custom head mount by dental cement. Body temperature was monitored and maintained at 37°C with a heating pad. After local application of bupivacaine (8 mg/kg), the skin covering the dorsal skull was removed. The bone overlying the olfactory bulbs was thinned by a dental drill and miniature scalpel. Vaseline was applied to form a well on the bone around the cranial window. The window was filled with saline and sealed with a cover glass. Anesthesia was maintained throughout experiments by intraperitoneal administration of ketamine and xylazine via a cannula inserted into the abdomen.

### Optical imaging

To characterize glomerular responses to retronasal odor stimuli, odor responses in the dorsal olfactory bulb were measured *in vivo* through a cranial window created by thinning the overlying bone [40]. Imaging was performed using an Olympus BX50WI microscope equipped with a 4× (0.28 NA) objective. The dorsal surface of the left olfactory bulb was illuminated with 480 ± 20 nm light using a collimated cyan LED (LEDC9, Thorlabs) and a 505 nm long-pass dichroic mirror, and fluorescence emission above 510 nm was collected. In each recording, data were collected for 60 s at 10 Hz. Odor stimuli were applied starting at 10 s after start of recording. Images were acquired using Image Pro 7.0 software (Media Cybernetics) and a Photometrics Cascade II 512B EM-CCD camera with a resolution of 256 × 256 pixels. The imaging area was 2.1 × 2.1 mm.

### Odorant stimulation

Odorants (butanal, butyric acid, eugenol, heptanal, heptanoic acid, hexanal, hexanoic acid, methyl benzoate, methyl isoeugenol, octanal, pentanal, propionic acid, valeric acid; all 95 – 99% purity) were purchased from Sigma-Aldrich. Odorant stimuli were prepared in 250 ml amber glass bottles, either as a neat substance or diluted in mineral oil. Odorant concentrations are expressed as percent liquid dilution of pure odorant. Odorant vapor in the headspace of a bottle was delivered by a 12-channel olfactometer. A separate channel was used for each odorant to avoid cross-contamination. Continuous clean airflow was replaced by odorant airflow for the duration of a stimulus pulse. The inter-stimulus interval was at least 2 min. Each odor stimulus was repeated 3 times. The olfactometer was controlled by a program written in LabVIEW (National Instruments, Austin, Texas). A schematic diagram of orthonasal and retronasal odor presentation is shown in Figure 1. For orthonasal stimulation (Figure 1A), odorized air was delivered externally in front of the mouse’ nose at a rate of 1,500 ml /min for 10 s or 30 s through the nozzle. Suction was constantly applied through the upper tracheal cannula at 150 ml/min by a vacuum pump. In the orthonasal mode, the flow rate in the nasal cavity was set by a flow meter connected to the vacuum pump. For retronasal stimulation (Figure 1B), odorized air was delivered from the upper tracheal cannula at 150 or 300 ml/min for 10 or 30 s by the olfactometer. Suction was not applied in the retronasal mode. Stimulation modes could be switched reciprocally in the same animal preparation.

### Data analysis

Data processing was performed using Image Pro 7.0 software. To generate glomerular maps of odor-evoked spatial activity, time sequence data of spH signals were normalized and corrected for photobleaching. The odor-evoked change in spH fluorescence (ΔF) was calculated at each pixel by subtracting the temporal average over a time window preceding the stimulus (*t*_1_) from the temporal average centered on the peak of the response (*t*_2_). SpH signals during the period 7 – 9 s were averaged for *t*_1_, regardless of duration of odor pulses. The period of *t*_*2*_ was 20 – 22 s in the case of 10 s stimulus duration. When odor stimulus was presented for 30 s, the period of *t*_*2*_ was 38 – 40 s. The relative change in fluorescence (ΔF/F) was then calculated by dividing the odor-evoked change in fluorescence (ΔF) by the resting fluorescence at time window *t*_1_, and rendered in pseudocolor. Each spatial map was smoothed by a Gaussian filter (3 × 3 pixel kernel) and 3 maps from each odor stimulus were averaged for the generation of figures. For quantitative analysis of individual glomeruli, the spH signal amplitude was analyzed by spatially averaging pixels overlying the glomerulus of interest. Response amplitudes were averaged over 3 trials to obtain a final mean ΔF/F value of each glomerulus for each odor stimulus. Data are expressed as mean ± SEM. For the time-course of spH signals, raw traces were corrected for photobleaching by subtracting trials in which no odor stimulus was given. Response traces were then calculated and averaged from the ΔF/F response of 3 trials measured at pixels overlying a glomerulus. Response traces normalized to the maximum response amplitude are shown in Figure 2.

## Abbreviations

EOG: Electroolfactogram; OR: Olfactory receptor; OSN: Olfactory sensory neuron; spH: SynaptopHluorin; Log P: Log octanol/ water partition coefficient; SEM: Standard error of the mean.

## Competing interests

The authors declare the following competing interest: the research described in this study and the publication of this manuscript were funded by a grant from Japan Tobacco Inc.

## Authors’ contributions

YF designed and carried out the experiments, data analysis and wrote a draft of the manuscript; GC and GL constructed the olfactometer; GL supervised the study and participated in its design, coordination, manuscript editing and completion; all authors read and approved the final manuscript.
